# Myoglobin removal of small-protein leakage membrane (EMIC2) in patients in the ICU: a case series

**DOI:** 10.1186/cc13588

**Published:** 2014-03-17

**Authors:** P Fabbrini, R Rona, M Migliari, M Viganò, A Pesenti, A Stella

**Affiliations:** 1 AO San Gerardo, Monza, Italy; 2 Università degliStudi Milano-Bicocca, Milan, Italy

## Introduction

Rhabdomyolysis is characterized by breakdown of striated muscle due to a great number of causes. Acute kidney injury (AKI) is a common complication as a consequence of high concentrations of circulating myoglobin (Mb). The AKI degree can vary but often requires dialysis, a condition which drastically worsens the ICU stay and prognosis. Since Mb overconcentration represents the cause of AKI, one of the therapy's aims should be its removal to prevent further kidney damage and to allow faster renal recovery. Both intermittent hemodialysis and high-volume CVVHF are poorly effective in removing Mb, while small-protein leakage membranes seem to be promising in this setting. The aim of our study was therefore to measure efficacy of Mb removal of a new high cutoff membrane (EMIC2; Fresenius, cutoff value 40 kDa) for continuous renal replacement therapies (CRRT) in the ICU setting.

## Methods

We report results of EMIC2-based treatments in seven patients (four male/three female) with different causes of rhabdomyolysis (trauma, sepsis, limb ischemia). Five patients had classic dialysis indications (persistent anuria) while in two patients treatment was prophylactically started. CRRT were delivered in CVVHD mode with the EMIC2 dialyzer and with loco-regional trisodium-citrate anticoagulation. Mb plasma levels were assessed each 12 hours while the removal rate, total body and dialyzer clearances were estimated by kinetic modeling as previously described [1]. Clinical data were also collected and both global and renal patient survival was reported.

## Results

The median Mb value at CRRT start was 6,971 ng/ml (range 4,679 to 48,011 ng/ml). CRRT were delivered with an average blood flow rate of 143 ± 45 ml/minute and a dialysate flow rate of 2,134 ± 1,334 ml/hour. These operating conditions allowed one to stop treatment on average after 75 ± 47 hours (median 54 hours) with a Mb reduction of 82.2% (range 99.4 to 44.4%). Overall median Mb removal per treatment was 59 mg (range 33 to 279 mg) mainly due to the first 24 hours of treatment (54 mg, range 20 to 187 mg). Only two patients had residual renal function that was in one case measured to account for only 7.45 mg Mb removal during the entire treatment. Six patients survived and recovered renal function with no dialysis need at present follow-up. One patient died during the ICU stay.

## Conclusion

Our data measured high performance of the EMIC2 membrane in Mb removal and confirm theoretical models indicating that CRRT with a high cutoff membrane can achieve major Mb removal within 24 hours with great superiority in comparison with all other available techniques.
